# Pregnant women’s experiences with an integrated diagnostic and decision support device for antenatal care in Ghana

**DOI:** 10.1186/s12884-018-1853-7

**Published:** 2018-06-05

**Authors:** Ibukun-Oluwa Omolade Abejirinde, Renate Douwes, Azucena Bardají, Rudolf Abugnaba-Abanga, Marjolein Zweekhorst, Jos van Roosmalen, Vincent De Brouwere

**Affiliations:** 1 Athena Institute, Faculty of Science, Vrije Universiteit, Amsterdam, The Netherlands; 2 Department of Public Health, Institute of Tropical Medicine, Maternal and Reproductive Health Unit, Antwerp, Belgium; 3 ISGlobal, Hospital Clínic- Universitat de Barcelona, Barcelona, Spain; 4 Simavi, Amsterdam, The Netherlands; 5 Presbyterian Health Services-North, Bolgatanga, Upper East Region, Ghana; 6 Department of Obstetrics, Leiden University Medical Centre, Leiden, The Netherlands

**Keywords:** mHealth, Antenatal, Woman-provider interaction, Prenatal screening, Ghana, Clinical decision support

## Abstract

**Background:**

Quality antenatal care (ANC) is recognised as an opportunity for screening and early identification of pregnancy-related complications. In rural Ghana, challenges with access to diagnostic services demotivate women from ANC attendance and referral compliance, leading to absent or late identification and management of high-risk women. In 2016, an integrated diagnostic and clinical decision support system tagged ‘Bliss4Midwives’ (B4M), was piloted in Northern Ghana. The device facilitated non-invasive screening of pre-eclampsia, gestational diabetes and anaemia at the point-of-care. This study aimed to explore the experiences of pregnant women with B4M, and its influence on service utilisation (“pull effect”) and woman-provider relationships (“woman engagement”).

**Methods:**

Through an embedded study design, qualitative methods including individual semi-structured interviews and non-participant observation were employed. Interviews were conducted with 20 pregnant women and 10 health workers, supplemented by ANC observations in intervention facilities. Secondary data on ANC registrations over a one-year period were extracted from health facility records to support findings on the perceived influence of B4M on service utilisation.

**Results:**

Women’s first impressions of the device were mostly emotive (excitement, fear), but sometimes neutral. Although it is inconclusive whether B4M increased ANC registration, pregnant women generally valued the availability of diagnostic services at the point-of-care. Additionally, by fostering some level of engagement, the intervention made women feel listened to and cared for. Process outcomes of the B4M encounter also showed that it was perceived as improving the skills and knowledge of the health worker, which facilitated trust in diagnostic recommendations and was therefore believed to motivate referral compliance.

**Conclusions:**

This study suggests that mHealth diagnostic and decision support devices enhance woman engagement and trust in health workers skills. There is need for further inquiry into how these interventions influence maternal health service utilization and women’s expectations of pregnancy care.

**Electronic supplementary material:**

The online version of this article (10.1186/s12884-018-1853-7) contains supplementary material, which is available to authorized users.

## Background

Worldwide, significant progress has been made to reduce maternal mortality and morbidity. However, many low and middle-income countries (LMICs) remain significantly burdened by preventable maternal deaths and pregnancy-related morbidities [1, 2]. Substantive efforts to improve maternal health globally is therefore a key agenda under the Sustainable Development Goals [3, 4]. Furthermore, there is an observed shift from higher proportions of maternal deaths due to direct causes such as haemorrhage and sepsis, to increasing mortality related to indirect causes such as heart disease and anaemia, i.e. the obstetric transition [5, 6].

Quality antenatal care (ANC) is widely recognised as an important opportunity for screening and early identification of pregnancy-related complications such as preeclampsia, anaemia, and gestational diabetes [7, 8]. In cases of late or failure to diagnose, these conditions may cause severe morbidity and mortality. It is estimated that haemorrhage, often in combination with anaemia, as well as preeclampsia, account for about 27 and 14% of all maternal deaths worldwide [9, 10]. Specifically, in Ghana, the Maternal Mortality Ratio (MMR) in 2015 was reported at 319 per 100,000 live births with a large range of uncertainty (from 216 to 458) [11]. Haemorrhage (39%), hypertensive disorders (35%) and unsafe abortions (7%) were the main direct causes, while indirect causes (26%) include severe anaemia, diabetes and malaria [12].

In rural and remote areas of Ghana, comprehensive ANC is often hampered by staff shortages, lack of diagnostic equipment and supplies or weak referral linkages [13, 14]. Pregnant women are therefore often referred to distant health facilities, laboratories or private facilities for routine diagnostics. The resultant loss of time and money required to visit these facilities, demotivate women from ANC attendance and referral compliance, and may result in late identification and management of pregnancy-related complications.

Mobile Health (mHealth) solutions, defined as “the provision of health services and information via mobile technologies” [15] have been reported as beneficial for improving ANC services in LMICs [16–18]. There is a growing body of literature stating that mHealth solutions can potentially reduce gaps and inefficiencies in health service delivery including point-of-care screening, integration of records and streamlining care processes [19–22]. However, there is sparse evidence on the influence of such interventions on service utilisation and woman engagement. Using a mHealth intervention in rural Ghana as a case study, this paper aims to address this gap.

In 2016, a one-year proof-of-concept study tagged Bliss4Midwives (B4M) was launched in two regions of Northern Ghana- Upper East and Northern regions, with a goal to improve ANC services. The B4M device is an integrated diagnostic and Clinical Decision Support System (CDSS) that enables non-invasive point-of-care screening for pre-eclampsia, gestational diabetes and anaemia. B4M components include a non-invasive device for measuring haemoglobin via infrared sensors mounted on a finger clip; a self-inflating blood pressure cuff; and an automated reader for urinary protein and glucose through dipsticks. Data from all diagnostic devices are automatically or manually linked to an android tablet equipped with decision support algorithms. Additionally, a traffic light system (red, orange and yellow colours) visually indicates risk category or referral urgency for pre-eclampsia, gestational diabetes and anaemia, while prompting the health worker on counselling and treatment. Before commencing the pilot project, users (midwives and community health nurses) were trained on the technical and operational functions of the device. Training also included modules on the principles of quality ANC and management of pregnancy complications.

The project hypothesised that through this mHealth-health worker interface, the interpersonal process of care- a component of quality ANC- would improve. It was also expected that coupled with treatment or referral advice, the traffic light signalling would increase women’s engagement with the midwife, and women’s compliance. Additionally, by being responsive to the needs of pregnant women for screening tests, the project anticipated that use of B4M would increase demand and utilisation of ANC services. Summarily, the pilot project offered the opportunity to assess the influence of mHealth on ANC demand or service utilization (“pull effect”), and on the interpersonal process of care (“woman engagement”).

This paper therefore explores the experiences of women exposed to the B4M device, to answer the research questions: i) How did women experience the use of Bliss4Midwives during their routine antenatal care consultations? ii) What influence did Bliss4Midwives have on woman-provider relationships and on ANC service utilization?

Due to inactive use of B4M devices in the Northern region at the time of data collection, only findings in the Upper East region are reported.

## Methods

### Design

Qualitative methods including individual semi-structured interviews and non-participant observation were employed. Data collection was embedded into a broader realist evaluation of midwives’ adoption and utilization of the B4M device.

### Setting

The Upper East region (UER) is one of the 10 administrative regions in Ghana and is further sub-divided into districts and municipalities. The B4M project was piloted in Bawku municipal (one health facility- identified as Facility A) and Binduri district (three health facilities- identified as B, C and D) in the UER. Both Bawku municipal and Binduri district are predominantly rural and about half of their population is illiterate [23, 24]. Three B4M devices were placed in health facilities on a fixed (permanently stationed in facilities A and D) or rotational basis (one device shared by facilities B and C) and used to screen women attending ANC.

### Data collection

Exit interviews following ANC screening were conducted using a semi-structured interview guide. Questions explored women’s initial reactions to the device, its perceived benefits, their views on its (potential) effect on ANC uptake behaviour (i.e. pull effect), quality of service delivery and their desire for continued use of the device.

Interview guides for pregnant women were developed in English language and translated to the local language- Kusaal (see Additional file 1). These were discussed with local program managers before piloting and subsequent modification (ambiguous words identified and refined, exclusion of questions perceived to be culturally inappropriate). The first author and a trained female Research Assistant (RA) who is fluent in both English and Kusaal conducted 20 interviews with pregnant women in June 2017. Sixteen exit interviews were conducted in the four health facilities immediately after ANC visits with B4M use. Using disaggregated data from the project database and with the help of health facility staff, additional women who had been exposed to the device during past ANC visits were traced at community level and invited to the health facility for retrospective interviews (*n* = 4). Respondents were selected by convenience sampling based on their attending ANC at the health facility during data collection, or based on previous ANC screening with the device and availability for interview. Women were interviewed irrespective of gestation and type of ANC visit (i.e. first or follow-up). Interviews were conducted in locations close to health facilities, but not in the immediate vicinity of ANC consultations. Based on the level of education and preference of the respondent, interviews with pregnant women were conducted in English or Kusaal. Depending on the nature and unique circumstances related to timing and respondents’ convenience, the RA was sometimes the main interviewer while the principal researcher observed or functioned as the main interviewer, with the RA translating.

In addition, 10 semi-structured interviews in English were conducted with health workers (midwives and community health nurses) who operated the B4M device in intervention facilities and thereby were engaged in the B4M-ANC care process (see Additional file 2). This was done to understand the experience of use and nature of the mHealth-mediated consultation from different actors. Health workers were asked to share their perceptions of the influence of B4M on women’s behaviour, compliance to referral or clinical recommendations, and on women’s attitudes to the use of mHealth for ANC. As part of the realist evaluation in which this study was embedded, workers were asked to respond to a colour-guided Likert five-point scale (ranging from ‘strongly disagree’ to ‘strongly agree’) in response to specific questions about B4M use. The questionnaire section of the interview explored perceptions of how women responded to the device. Specifically: *“I think more pregnant women are coming for ANC now that we use B4M in the Health Facility”* and *“I think that pregnant women follow my advice more now that I use B4M for consultation.”* Responses to these questions were therefore included in the analysis for this paper. We aimed to interview all health workers who had been trained in B4M use, had used the device at least once post-training and were available at the time of data collection. Due to staff rotations, not all midwives who were initially trained were available to be interviewed and not all health workers in each facility were trained on the use of B4M. A summary of the number and category of interviews is presented in Table 1.

**Table 1:** Categories of respondents per health facility

	Health Workers	Pregnant Women	
		Exit interviews	Retrospective interviews
Health Facility^a^			
Facility A	3	6	0
Facility B	2	7	0
Facility C	3	0	1
Facility D	2	3	3
TOTAL	10	16	4

^a^Facility A is a district hospital in Bawku Municipality and is the first level referral point for facilities B, C and D which are health centers in Binduri district

All interviews were audio recorded and later transcribed and translated where necessary to English language. Two independent individuals transcribed a random number of interviews done in Kusaal to assure the quality of transcription and translation. Duration of interviews with pregnant women was between 11 and 34 min (median 21.36 min). Because interviews with health workers were part of a broader evaluation objective, these lasted longer- from 35 to 91 min (median 52.29 min).

In order to triangulate and validate findings from interviews, one researcher using a semi-structured checklist and observation guide conducted non-participant observations of ANC consultations in three intervention sites (facilities A, B and D). These were documented using handwritten notes. Non-participant observation is stated here to mean that the researcher is not an active participant in the facility, but interacts occasionally with the people in a non-intrusive way through questions and active listening, if the opportunity presents itself [25].

Secondary data on first ANC visits (i.e. registrations) over a one-year period- from December 2015 to December 2016 (i.e. 6 months before and 6 months after the pilot commenced in June 2016) were extracted from health facility records to supplement findings on the perceived pull effect of B4M.

### Data analysis

All transcripts were read and a preliminary codebook was developed guided by the main themes explored in the interviews. Two researchers developed codes inductively. One researcher coded all transcripts while another coded a random number of six transcripts to test consistency of codes and support data analysis. Codes were clustered into themes based on similar or recurring patterns. Qualitative data analysis was supported using NVivo qualitative data analysis Software; QSR International Pty Ltd. Version 11, 2014.

### Ethical considerations

Approval for this study was granted by the Navrongo Health Research Centre Institutional Review Board (Approval ID: NHRCIRB18) and the EMGO+ Scientific Committee of the Amsterdam Public Health Institute (Reference Number: WC2017–026). Verbal and or written consent was secured from all respondents. Consent was secured prior to all interviews, and respondents signed or appended their thumbprints to an informed consent form. Respondents for exit interviews received transport reimbursement (equivalent value of USD$1 - $2). Health workers were not reimbursed since interviews were conducted at their work place.

## Results

Between June 2016 and April 2017, 950 ANC screenings (including follow-up visits) were conducted with the B4M device in the UER; 284 screenings from health facility A, 69 in health facility B, 129 in C and 468 in D. Socio-demographic characteristics of interviewees are presented in Table 2.

**Table 2:** Socio-demographic characteristics of respondents

Characteristic	Pregnant women (*n* = 20)	Health workers (*n* = 10)
*Age*		
*< 18*	1	0
18–24	8	1
25–29	3	2
30–34	1	5
35–39	2	1
40+	1	1
Missing data	4	0
*Number of Births*		
0–1	12	
2–3	4	
4–5	2	
Missing data	2	
*ANC Observations Conducted*		
Yes	14	
No	6	
*Cadre*		
Midwife		8
Enrolled Nurse ^a^		1
Community Health nurse		1
Years of Experience		
0–4		4
5–9		5
> 10		1

^a^An auxiliary cadre similar to health assistants

### Women’s first impressions of B4M

Pregnant women’s first impressions of the Bliss4Midwives device ranged from fear and happiness to curiosity or a neutral disposition (Table 3). The non-invasive Haemoglobin clip required women to insert an index finger into the peg-like clip and was a main reason for fear. This was because some beneficiaries did not know what to anticipate on insertion. Two women specifically reported a feeling of discomfort during the screening*.*

**Table 3:** Initial reactions of ANC attendees to B4M device

Reaction	Supporting Quote
Fear	*“It was because I had never seen it and that was my very first time of seeing it, so I was afraid”* -Pregnant woman 1, Facility D
Happiness	*“I was excited because I was hopeful that if I have any sickness in my system, the machine will let me know.”* -Pregnant woman 3, Facility A *“I would say for us here, when you hear that you have a device that is going to help you do something, they get excited…. some (women) get excited, but others…. it is the explanation that you have given them of what (the box) can do so that they can accept it.”* -HCW 3, Facility A
Neutral	*“I wasn’t happy and wasn’t afraid as well, I just knew it was part of the care they were providing”* -Pregnant woman 6, Facility B *“I thought using the box is only when it becomes necessary. So whether or not she uses it, it is the same… They are the professionals and know the best for me. As for me, I cannot tell.”* -Pregnant woman 1, Facility A
Curiosity	*“That, because after she said she was going to use it for this particular thing, when she was doing it, I was eager and ready to see whether I belong to any of the diseases she has mentioned or I’m free, so I was just waiting to see what the machine will tell me”* -Pregnant woman 2, Facility B

Happiness was related to technological novelty or appreciation for easier and additional diagnostic services. As noted during ANC observations, some attendees were neutral- rarely questioning or initiating conversations with the health worker, and were passive actors in the consultation process. Notably, the few women who demonstrated curiosity were formally educated (completed basic primary education). Health workers also corroborated reports of happiness and fear.

The ability of providers to clearly explain the device function and help women understand its benefits facilitated a positive disposition to B4M. Attempts by health workers to incorporate use of B4M while handling a high volume of work in understaffed settings, sometimes resulted in situations where not all women attending ANC were screened with B4M. An unintended consequence of this adaptation strategy was that while some women felt special for being selected, others felt otherwise excluded or came up with plausible explanations of why they were not screened with the device.

### Perceived benefits of B4M

ANC respondents felt satisfied about the services offered at the facilities. When asked to rate the quality of their just concluded ANC visit on a scale of 1–10, most respondents gave scores between 6 and 10 points with specific credit given to counselling and staff attitude (e.g. health workers are friendly and not shouting). However, these ratings could not be directly attributed to the B4M device, although a few respondents specifically stated that their satisfaction of the visit was because of the otherwise absent diagnostic service provided by B4M. Analysis showed that pregnant women appreciated the device for detecting their health problems and saving time and money that would have been otherwise expended on diagnostic referrals. Some women also believed the device improved the knowledge of health workers and made them pay closer attention to specific aspects of the woman’s health (Table 4).

**Table 4:** Pregnant women’s Perceptions of B4M

Factor	Supporting Quote
Service Provision	*“(I like) every part of the machine. I like it because the machine will tell you the right BP…. and then it will tell you the types of disease or the problems you are finding in your system.”* -Pregnant woman 2, Facility B
Improved Knowledge	*“(I think the midwife has more knowledge). Because it is through those things that she used to get the information about my condition. If not, (the results I got from) outside, she could have recorded it and give me the card to go away, but because of the machine she has took her time to also take me through this procedure to know whether it is really true that I have this disease and …she is sure that what they brought outside is comparing to her machine.”* - Pregnant woman 2, Facility B
Efficiency	*“It has helped to make her efficient. It is because the things we could have gone to the lab to do that requires so much time, this one is faster and easier.”* -Pregnant woman 3, Facility A
Time delays	*“I: So in your opinion which particular place do you spend a lot of time?* *R: At the machine* *I: So where do you think when worked on, will help reduce the time spent? At the records, the machine, the palpation area, the dispensary?* *R: No, just at the machine* *I: ….so the machine is what causes the delay* *R: Yes”* -Pregnant woman, Facility D

Prior to the intervention, diagnostic screening was not frequently conducted at the point of care and now constituted an additional task in the ANC workflow, necessitating more time. For this reason, a drawback on the perceived benefits of B4M use was that the ANC process took longer time, which made women impatient. Health workers interpreted this impatience to mean that women were not accepting the device. Reports on time estimates and perceived delays with B4M use ranged from 10 to 30 min extra as compared to the standard workflow. Technical difficulties (software freezes, slow response time), and procedural issues (low user dexterity with operating the device) were two main factors that contributed to delays. While there were reports of rare occasions where one or two respondents voiced their displeasure about time delays, midwives more commonly observed signs of displeasure through body language. One midwife mentioned an extreme case where the turnaround time was too long, and a woman got restless and left the facility.

Interestingly, while ANC attendees in the district hospital (facility A) were particularly grateful to B4M for saving time, their peers in the health centres did not make this association. Contrarily, up to two women from health centres B, C and D associated the use of B4M with a time delay. Observations revealed that the reported time saving benefits of B4M from respondents in facility A was connected to women otherwise having to spend up to 2 hours at the hospital laboratory to process routine tests. Except where other laboratory tests had to be conducted, B4M therefore became a welcome time-efficient alternative in the larger facility.

ANC workflow is usually structured in stations such that women have to move from one point to another for specific tests or different aspects of the consultation (palpation, updating records, weight, height and blood pressure check, collection of routine drugs). In this context, workers felt the integrated function of B4M made ANC routine easier and more comfortable. However, one minor but crucial deviation from the perception that B4M facilitated focused antenatal care was noted in observation visits showing that workflow was still broken when women needed to provide their urine samples. This usually necessitated going to an outhouse, which prolonged consultation time and was sometimes frowned upon by other workers within the facility who saw this as an interruption of the general workflow.

In health facilities where workers helped women (re)negotiate their ANC experience and workflow, women better understood the value and benefits of the box and this improved acceptability while diminishing complaints about time delays. As one health worker put it *“they develop some love in it and every time they come, even if it is wasting time, they will still wait”-* an indication that women may have also adjusted their expectations and factored in the extra time needed for use of the device.

### Women’s engagement with B4M

Prior to using B4M for the first time, the project required that health workers informed women about the device using a structured information sheet. Following this, the woman was required to confirm consent by appending a thumbprint or signature. This mandatory step served as an opening for interactive communication, but was not always carried out. Observations showed that not all women received a full explanation about the device, its functions and the procedures for the screening. On the other hand, the structured format of B4M compelled prolonged woman-provider interaction leading to improved counselling that was otherwise previously rushed or absent.

Observations and follow-up questions to workers revealed that most women were indifferent about the details of care and did not necessarily want to be involved. Women were reportedly more keen to receive assurances about their health status without spending much time at the facility. In two of the three facilities where B4M use was observed, the physical positioning of the device relative to the woman and B4M user prohibited (in the case of opposite seating) or was suboptimal for (e.g. screen not within field of vision) visual engagement by the woman.

With a few exceptions, midwives would usually explain the summary of the B4M encounter to the woman after screening and use the tests results as an opportunity to educate them on diet, birth preparedness and pregnancy care prior to scheduling the follow-up appointment. Although this partly explained why the B4M encounter took longer, pregnant women also felt listened to and more included in the care process.

*“Because I saw that everything, everything was ok. But that place (i.e. another health facility) I can’t see. But here, everything is plain and I will know that… I can see it. I see what are the problems, what they are advising me on and other things.”* -Pregnant woman 6, Facility D.

### Trust and compliance with B4M

Prior to project implementation, it was found that apart from challenges in the health system with referral linkages, women would often not comply with referral recommendations. Reasons for this included doubt in the severity of their problem, concerns about cost and time implications, and fear of being unattended or getting lost in an unfamiliar large health facility.

One of the B4M intervention hypotheses was that the traffic-light module of the system (which should be visible to the woman during ANC consultations) would facilitate women’s engagement and motivate referral compliance. Interviews explored the confidence of women in the diagnostic decisions from B4M and the possibility that women were more assured about the validity and necessity of referral instructions if it came from a digital third party. Analysis showed that most women were confident with the screening results and a couple of respondents noted that they would not have believed a referral recommendation if it had not come from the device.

*“Using the box makes me actually believe that it is indeed true, because with the box she is telling me what she has seen”* -Pregnant woman 7, Facility B.

However, there were a few reports of women who while acknowledging benefits of the device, did not necessarily trust its decisions or recommendations. In negotiating this digital trust, educated respondents were found most likely to critically reflect on results and recommendations from B4M.

All health workers agreed to varying extents (eight strongly agreed and two slightly agreed) to the question *“I think pregnant women follow my advice more now that I used the box for consultation”*. Reasons included the perception that women’s respect for workers increased- an extension of additional skills acquired through B4M. Many health workers felt that women’s compliance to referral and counselling recommendations happened because the machine, which was seen as a more knowledgeable or accurate medium, was ‘demanding’ it.

Some health workers were also of the opinion that the device mediated woman-provider relationship by enhancing trust.

*“Even here the moment you work with them and they have trust with you, that is all. They believe and they trust whatever you tell them…. And with the box, it has even enhanced our work. They believe and they trust that if we tell them something, it is true because the box has actually said it.”* -HCW 2, Facility D.

Interestingly, some women personified the device, reporting that the box “advised them” while others described what “the machine said”. Just as the receipt of routine drugs (iron and folate) at the end of a visit was considered proof that ANC had been effectively carried out, two pregnant women said interaction with the box was proof that ANC was properly completed.

*“You know when I come and they don’t use it to attend to me and I just get back home like that, it doesn’t actually feel like you came”* -Pregnant woman 3, Facility B.

### Pull effect

An anticipated effect of B4M use in health facilities was that its innovativeness and the provision of diagnostic options for women would encourage more first time (registrants) and follow-up ANC visits (attendants). Interviews revealed that all, except two health workers, felt that the use of B4M in their health facility created a demand for ANC services. In some cases, it was believed that B4M attracted women from other health centres to theirs. In response to the Likert-scale question *“I think that more pregnant women are coming for ANC now that we have this device in our health facility”*, two health workers neither agreed nor disagreed, three slightly agreed and five were strongly in agreement. A minority, however, felt that more women were now registering for ANC, but that this was unrelated to B4M.

Health workers attributed this perception of a positive pull effect to women spreading news about the device within their communities, especially following a testimony of improved wellbeing after adhering to the B4M advice.

*“When they saw it the first time and those (I screened) and told them their problem, they were telling their colleagues- ‘Oh I went to the hospital, they are now having a machine’. So everybody wants to come and see the machine.”* -HCW 2, Facility B.

However, no pregnant woman reported discussing the availability of the device with any of their peers or learning about its presence in the facilities from a third party.

Although we did not have enough data points to determine the extent to which number of ANC visits was associated with the presence of B4M in health facilities, we compared descriptive data on ANC registrations (1st visits) in UER intervention facilities 6 months prior to B4M (December 2015–May 2016) and 6 months following commencement of the pilot (July 2016–December 2016). Interestingly, results did not show a dramatic increase in total visit numbers. In some instances, we noticed a decline over time (Table 5).

**Table 5:** Number of ANC registrants 6-months pre- and 6-months post- B4M initiation^a^

	Facility A^b^	Facility B	Facility C	Facility D
Dec 2015- May 2016	792	199	79	171
July 2016- Dec 2016	647	205	88	135
Difference	− 145	+ 6	+ 9	−36

^a^Data from June 2016 was not included in this analysis because the intervention commenced in the middle of the month

^b^Facility A is a district hospital in Bawku Municipality and is the first level referral point for facilities B, C and D which are health centers in Binduri district

When women were asked to comment on their disposition to return for ANC even in the absence of the box, all noted that it would not influence their attendance of follow-up visits, although they may be curious or slightly disappointed if it was absent. This may have been due to the lack of alternatives as pointed out by one respondent:

*“Of course I will (keep visiting this health facility). Where else will I go? I was even coming here when the box was not here”* - Client 1, Facility B.

Although most women felt their future ANC behaviour would not be motivated by the presence of the device, their expectation for future use was affirmative.

## Discussion

This paper aimed to explore the experiences of pregnant women screened with B4M device and its influence on ANC service utilization and woman-provider relationships. There has been significant focus on the feasibility and acceptability of mHealth from the lens of the technology user. However, less attention has been paid to the views and perceptions of the end beneficiary- perspectives which are equally important in the outcome and long-term use of mHealth devices [26].

The reported enthusiasm of women to B4M can be linked to the novelty effect of mHealth and are similar to reactions of health worker users in other studies [21, 27]. While there are mHealth innovations where the user is the direct beneficiary of the functions offered [16, 28], the nature and function of B4M necessitated a triad relationship between the device, the health worker user and the direct beneficiary i.e. pregnant women. Findings that some women were initially fearful of the device shed light on the importance of incorporating both technology users and beneficiaries in the mHealth-assimilation process. Specifically, reactions of fear in the B4M study were directly related to device components, which can be overcome with careful explanation and guidance from health workers. The potential risk of skipping this process of careful negotiation is the spread of myths and misconceptions, which could hamper eventual integration of mHealth into routine care.

The neutral disposition of some pregnant women to B4M may have been related to low literacy levels and feelings of inadequacy about questioning the competence of a trained professional. Given the intervention context- predominantly rural with high illiteracy levels, it was not surprising that only a couple of B4M beneficiaries who are also educated, were engaged actively in the care process [29]. Future interventions of a similar nature should go beyond technical training; additional training in communication and negotiation skills is necessary to facilitate acceptability and to actively involve women in the care process.

This study found that although pregnant women could not fully engage with the B4M-mediated process, they were still satisfied with the care received. The design of B4M compelled health workers to follow structured guidelines and procedures, such that they had to directly communicate with women. In so doing, the device served as a platform for improved woman-provider interaction. This made women feel heard and listened to, although an indirect consequence of this was longer consultation time. Missed opportunities for improved engagement occurred when workers did not take the entry point offered by the intervention to explain its function to end-beneficiaries. By virtue of providing diagnostic services, which would otherwise take a longer time or incur additional time and financial commitments of pregnant women, B4M served to overcome gaps in unnecessary diagnostic referral while influencing the likelihood that women would comply with referral recommendations. A note of warning, however, is that if women become only compliant to clinical recommendations when delivered by technology, there is risk of a dilemma should woman-mHealth trust rank above woman-provider trust.

While technology has shown promise in improving access and efficiency of some health care processes, it is generally agreed that it is not yet a substitute for real-time face-to-face interaction and interpersonal relationship [30]. There are mixed reports on how mHealth may facilitate or hinder woman-provider interaction [30–32]. A study on the perceptions of community health workers in Brazil to the use of a data collection and transmission tool, reported social barriers to utilizing the cell phone intervention despite its reported advantages [31]. Such barriers included the negative impact of the tool on social interaction (i.e. interpersonal connection) with pregnant women. Interrupted eye contact and passive involvement especially with illiterate and older women impeded trust building and effective engagement. On the other hand, assessment of the *mobile santé Nouna* intervention in rural Burkina Faso showed expectations of improved relationships between the health facility and the community with mHealth as an intermediary [33].

The meanings of expertise that women attached to the B4M device while personifying it were notable. This made B4M seem like an active expert-participant in the consultation. A review of studies found that when beneficiaries of care attribute a status of specialised or “higher up” knowledge to CDSS, it enhances their trust in results and recommendations [21]. Woman-provider trust has been shown to play a role in the continuity of maternal care and in women’s preferences for facility delivery [34, 35] and ANC visits have been earmarked as an entryway to establish such trust [7]. Although some health workers believed that the B4M device mediated the woman-provider relationship by enhancing trust, we could not ascertain the pattern of interactions regarding the trust effect. Were ANC attendees trusting health workers more because they used the device, or did it only enhance existing trust? Especially with B4M where screening results required voluntary compliance from women to seek further care, their trust in test results and the midwife’s recommendations was crucial. Screening, which is only one of many steps in the chain of care is therefore only relevant when followed by prompt and proper management of the conditions detected. Unfortunately, broader health system deficiencies such as lack of resources and fragmented referral systems limit the capability for a full chain of service delivery, which is beyond the scope of what mHealth can do.

Interestingly, perceptions of time efficiency differed between types of health facility. The mixed reports about the time efficiency of B4M use in health centres (facilities B, C, D) and the hospital (facility A) appeared to be linked to the workload associated with the latter. B4M allowed women bypass burdensome bottlenecks of the care process in the bigger health facility. Studies have shown that service delivery advantages of mHealth vary depending on characteristics of the facility in which it is placed and aspect of care it is used for. Reports from a multi-country study show greater time efficiency when CDSS was used in the delivery room than for ANC [36]. This may explain why B4M was considered more time efficient at the district hospital than at lower levels where women had more direct contact with all points of the ANC workflow without too many interruptions in the care process. Overall, our findings show that B4M saves time compared to the standard where women from health centres have to delay screening and travel a distance for tests. In this standard scenario, ANC consultation becomes interrupted till a later date, after test results have been acquired. These are time inefficient processes that may not be immediately appreciated by pregnant women and overall influence the timeliness of treatment and continuity of care.

In the last decade, the effect of community focused information, education and communication campaigns about the value of ANC seems to have paid off. Even in rural areas of Northern Ghana, studies have reported impressive rates (up to 90%) of ANC attendance and utilization of skilled delivery [37]. While this study could not prove increased ANC registrations due to B4M use, health workers had a strong perception of a pull effect. The counter-intuitive results from B4M facilities with continuous use versus rotating use, where registrations marginally decreased in the former group compared to the latter, further caution against hasty conclusions regarding a pull effect and hint at other factors at play to explain these differences. However, some studies in similar settings have reported improvements in health service utilization, specifically ANC attendance, due to mHealth [28, 38, 39]. In addition to a small data set, the absence of agreement between workers’ perceptions and routine data from the B4M sites may be due to incomplete records, pre-existing trends of high ANC coverage in study regions, or minimal options at competing health facilities from which clients could be pulled. In light of updated guidelines recommending eight ANC contacts during pregnancy [7], and the continued expansion of mHealth use in LMICs, it is beneficial to identify the contexts and mechanisms by which mHealth positively influences demand and service utilization.

### Study limitations

The B4M intervention was a short-term proof of concept and as such its use was confined to a limited number of health facilities in study regions. Retrospective interviewing of some pregnant women as well as B4M users who had not used the device for up to 2 months before the interview, introduced a risk of recall bias. We attempted to counter this by using visual cues of the device and prompting respondents with specific events associated with device use. In addition we used multiple data sources for triangulation. By conducting women’s exit interviews in locations removed from the immediate service delivery environment, other biases (e.g. social desirability and confirmation) were minimized. A researcher who was not familiar with the local language conducted observations. This limited the ability to fully capture verbal elements of what was observed. However, when possible, information gaps were filled by follow-up questions to health workers. Research bias due to the researchers’ presence during the initial sets of observations was inevitable. To counter the observer effect, multiple observation visits were conducted over a number of days, with the expectation that those being observed will resume their natural behaviour over time [40]. Not all respondents declared their age and health record booklets were not readily accessible, leading to information gaps on socio-demographic characteristics.

## Conclusions

Pregnant women’s experiences and perceptions of mHealth-supported service delivery can influence how the implementation process translates to desired care outcomes. The B4M proof-of-concept showed that mHealth influences the trust ANC attendees have in health workers and in referral recommendations, which may positively impact compliance of women to treatment. Additionally, by fostering some level of engagement, the intervention made women feel listened to and cared for. There is, however, a need for further inquiry into how mHealth shapes women’s expectations of maternal care and the perceived pull effect. Future studies should also focus on how beneficiaries, in addition to mHealth users, directly or indirectly influence mHealth adoption, utilization and health outcomes.

## Additional files

Additional file 1: Question Guide: Client exit interviews. (DOCX 145 kb)

Additional file 2: Question Guide: Health worker interviews. (DOCX 141 kb)

## Abbreviations

ANC: Antenatal care
B4M: Bliss4Midwives
CDSS: Clinical decision support system
LMICs: Low and middle-income countries
mHealth: Mobile health
RA: Research assistant
UER: Upper East Region
