# Supplementary material for: Pregnant women’s experiences with an integrated diagnostic and decision support device for antenatal care in Ghana
Source: BMC Pregnancy Childbirth. 2018 Jun 5;18:209. doi: 10.1186/s12884-018-1853-7 (PMC5989381; doi:10.1186/s12884-018-1853-7)
Supplement: Supplementary file 1 — Question Guide: Client exit interviews. (DOCX 145 kb) [file 12884_2018_1853_MOESM1_ESM.docx]

**Question Guide: Client Exit Interviews**

**Introductions** (start with general greetings and ask about her well being)

1. Thank you so very much for agreeing to have this short discussion with me (*assure client of confidentiality, no right or wrong answers and the freedom to speak freely and ask questions as well*).

Please can you confirm for me again that you voluntarily agree to take part in this interview? *(Now you have consent on the recorder).*

1. My name is _________, please tell me your name and which village you come from?

How did you get here today? (*e.g. walk, motorbike, someone brought her, etc*) Is this how most women come to the health facility?

**Expectations and Perception of ANC visit**

1. How long has it been since you came to the health facility today? (***Probe:*** check for duration of ANC). Did it take longer than you expected? Or is it normal to spend this much (or this little) time when you come?
2. (*Ask client to walk you through her ANC visit from the time she arrived till now*). ***Note:*** *follow up her narration with questions about what was said to her, what tests were conducted, what drugs she was given, the advice she received and the conclusion of the consultation (e.g. next visit day or follow-up with more tests or treatment).*
3. Was there anything you were expecting would be done that was not done? (*e.g. test, examination, feedback/advice, counseling/education, other information*) (*Probe for clients’ unmet expectations?****)***
4. Did the midwife advice you about your health with respect to high blood pressure or anemia? *Ask for details*
5. Are you satisfied with the service you have received today? (***Ask****: very satisfied, satisfied or not satisfied*). ***Probe further*** *with the following questions*:
6. Do you think that the ANC you received from this facility is of good quality

(***Probe:*** *If yes: i) ask what she understands by good quality ANC? i.e. what are examples of resources or activities are indicative of quality ANC?* ii) *Ask her to score her perception of the quality of ANC that she has just received on a scale of 1 (minimum) to 10 (maximum)? iii) Ask her further to motivate her reason for this score; Ask if it has always been like this or quality recently improved).*

1. What specific aspects of the ANC consultation process would you like to see improvements in?
2. When you are attend ANC visits, which of these three is most important to you:

i) The manner in which the midwife speaks and interacts with you, by listening and answering your questions politely?

ii) The different procedures and tests she conducts?

iii) The amount of time you spend receiving ANC?

(***Note:*** *Multiple responses are allowed/ Ask why she considers them important*).

d) Did you have to pay for anything today? If yes, how much and for what?

**Bliss4Midwives Use & Perception**

(*Show picture of the B4M kit*) ***Ask:*** did you see this device today during your ANC? (*Yes or no?* ***If yes****, check if it was actually used on her.* ***If it was not used on her, skip to question 8***). *If woman confirms that the device was used on her, continue:*

1. What was your initial reaction or thought the first time or day you saw this box? ***Probe*** e*.g. fear, excitement, and worry. Ask why*

***Confirm when was the first time she saw it to check repeated exposure to the box or first time exposure.***

What do you believe the box does? ***Probe*** *for clients understanding of what the box does.*

1. Did the midwife explain what it is for and why she is using it? What exactly did she tell you?
2. What did you notice or observe while the box was being used on you? ***Ask client to walk/talk you through her thoughts and observations while the various processes were being carried out with the box.***
3. What was the outcome of the assessment after they used the box on you today? ***Probe*** *for clients perception and impression of the decision given by the midwife/the box.* ***A good way to ask this is: What did the box say at the end?***
4. Do you trust the information the midwife gave you after using the box? Why? Is it because of the box or do you trust the information and skills you get from the midwife even without the box? ***Probe: is trust due to the presence of the box or trust in the knowledge and skills of the midwife previously existed but are now further enhanced by the box?***

Did the use of the machine make you uncomfortable? ***Probe:*** *why or how exactly i.e. what about the device made you uncomfortable?*

Were you excited or happy that they used this device during ANC? ***Probe:*** *why or how exactly i.e. what about the device made you uncomfortable?*

1. Do you think the midwife has more skills or knowledge because of the box? *Why or why not?*
2. Did you like anything about the use of this device? *(****Probe:*** *what exactly and why did she like it?)*
3. Is there anything you did not like about the device? What? (***Probe*** e*.g. it takes too much time, it makes some strange sounds, it makes her uncomfortable).*
4. Have you attended ANC before where this device was not used? How long ago was that? Compared to that visit that did not involve the use of this device, in your opinion, which do you prefer (i.e. with device or without device) and why? (***Probe****: do you feel that you got better service with the use of the* device*? Why do you think so? E.g. the midwife counsels me better and talks to me more)*
5. Do you think other pregnant women will like this device? Why or Why not? ***Probe****: has she heard other clients making any kind of remarks about the device?*
6. How about you? Why would you like it to be continuously used? What benefits do you think you get with the box that you previously did not have? *(****Probe:*** *for trust, accuracy and reliability of information)*
7. Does this box influence (or have an effect) on if you will continue to come for ANC weigh in or it does it not matter if the box was not here. ***Probe:*** *did you maybe come for ANC because you heard they have this machine here?*

**Referral Linkages and Compliance**

1. How do you feel when the midwife tells you that you need to go to the Hospital to do more tests or to see a doctor? What are the first things that come to your mind? ***Probe for possible reactions:*** *fear, concern about distance, concern about the attitude of workers there, concern about the financial implication etc*
2. What are the challenges with seeking care at the main hospital? *(****Probe:*** *financial, distance, perception of low quality, socio-cultural etc.)*
3. Just to wrap up our discussion, please tell me what challenges do you think women in your community face with receiving quality antenatal care? *(Ask for specific examples of challenges)*
4. Do you have any questions for me, or would you like to share any additional information?

**Thank you very much for your time.**

*Ask respondent to sign/thumbprint informed consent sheet to confirm that she has received …… ghana cedis from you for her transportation costs.*
