# Supplementary material for: Pregnant women’s experiences with an integrated diagnostic and decision support device for antenatal care in Ghana
Source: BMC Pregnancy Childbirth. 2018 Jun 5;18:209. doi: 10.1186/s12884-018-1853-7 (PMC5989381; doi:10.1186/s12884-018-1853-7)
Supplement: Supplementary file 2 — Question Guide: Health worker interviews. (DOCX 141 kb) [file 12884_2018_1853_MOESM2_ESM.docx]

**Question Guide: In-depth Interviews, Bliss4Midwives (B4M) users**

**1) Personal Data**

a) Thank you very much for agreeing to have this short discussion with me. Please can you confirm for me again that you voluntarily agree to take part in this interview? *(Now we have consent on the recorder).* Thank you.

Please can you tell me how long you have been workings as an ANC provider?

b) How about the total number of years working as a health care professional?

c) And your cadre is? *(****Clarify:*** *Midwife; CHN, MO)*

d) Please tell me how old you are?

e) And what is your highest level of education?

f) How long have you been working in this health facility?

**2) Experiences of using B4M**

a) Before the B4M device was introduced in this health facility (i.e. before June 2016), how were you conducting ANC? *(****Probe:*** *What challenges with providing ANC were you facing then? Are you still facing those challenges up till now?)*

b) Do you think anything has changed since it was introduced? i.e. What difference (if any) has use of the system made to your work? *(****Probe:*** *e.g. to work process, professional satisfaction, relationship with clients etc. Clarify or ask for examples.* Why do you think it makes this difference? *(****Probe:*** *e.g. gives support, saves time, and improves confidence)*

c) What are you now able to do with B4M device that you could not do before it was brought to this health facility?

d) What were you able to do well before B4M was here but now that you have the device, it helps you to even do those things better? *(****Probe:*** *Why is this difference important to you?)*

*d)* Based on your overall experience with using the B4M system, do you feel you can carry out your ANC services work effectively without this system or do you consider it a necessity? *(****Probe:*** *In what way? Why?)*

*e) Can you give me some examples of some* factors or people that affect your ability to use the system- either that support you or hinder you from using the device?

*(****Probe*** *for range of facilitators and barriers. e.g. ability to charge the kit and charging duration? How many hours does it last for use (whole day); ANC attendance by women; Availability of consumables (Where do you get your consumables e.g. Urine dipstick?); Patient preference and acceptance of the system; no peer support; long waiting lines or general work overload; difficult to use)*

f) Do you think there are certain types of midwives for which this device will not be useful? *(****Probe:*** *e.g. based on age, years of experience, general disposition to technology etc.* ***Probe:*** *What is it about mentioned attributes that make the device not suitable or not useful to these midwives)?*

g) Do you think there are certain types of health facilities where this device will not be useful? *(****Probe:*** *e.g. based on available resources, staff mix and number, type of services offered, etc.* ***Probe:*** *what is it about mentioned attributes that make the device not suitable or not useful in these health facilities)?*

h) Do you think there are certain types of pregnant women who will not like the use of this device on them? *(****Probe:*** *e.g. based on personal reservations, and myths etc.)?*

i) Please tell me, in your opinion, which types of midwives are more likely to use this device?

j) In your opinion, which types of health facilities), (or pregnant women) do you think this device is more useful for?

*Administer questionnaire and continue interview afterwards*

**3) Third Party Reactions**

a) How do women react to the system during ANC consultations?

*(****Probe:*** *How do you think they feel about its use? Are there some who are resistant to the system? In what way?)*

b) Do you feel that when you use the device during ANC, your interaction with the women improves? *(****Probe****: Why do you say so? Can you give an example?)*

c) Do you feel that the women respect you more when they see you using the device? *(****Probe****: How exactly do you mean or why do you feel this way?)*

d) Do you think the B4M kit improves the referral system in any way? In what way?

*e)* Do you think the B4M kit helps to improve referral compliance by pregnant women? ***Probe:*** *What makes you say so?*

Do you have any questions for me, or would you like to share any additional information?

**Thank you**  *(Wrap up the interview*)
